# Distinct alterations of functional connectivity of the basal forebrain subregions in insomnia disorder

**DOI:** 10.3389/fpsyt.2022.1036997

**Published:** 2022-10-13

**Authors:** Guihua Jiang, Ying Feng, Meng Li, Hua Wen, Tianyue Wang, Yanan Shen, Ziwei Chen, Shumei Li

**Affiliations:** ^1^Department of Medical Imaging, Guangdong Second Provincial General Hospital, Guangzhou, China; ^2^Department of Radiology, Affiliated Hospital of Chengdu University, Chengdu, China; ^3^The First School of Clinical Medicine, Guangdong Medical University, Zhanjiang, China; ^4^Department of Medical Imaging, Guangdong Second Provincial General Hospital, Jinan University, Guangzhou, China

**Keywords:** functional connectivity, insomnia disorder, anxiety, basal forebrain, resting-state fMRI

## Abstract

**Background:**

Cholinergic basal forebrain (BF) plays an important role in sleep-wake regulation and is implicated in cortical arousal and activation. However, less is known currently regarding the abnormal BF-related neuronal circuit in human patients with insomnia disorder (ID). In this study, we aimed to explore alterations of functional connectivity (FC) in subregions of the BF and the relationships between FC alterations and sleep and mood measures in ID.

**Materials and methods:**

One hundred and two ID patients and ninety-six healthy controls (HC) were included in this study. Each subject underwent both resting-state fMRI and high-resolution anatomical scanning. All participants completed the sleep and mood questionnaires in ID patients. Voxel-based resting-state FC in each BF subregion (Ch_123 and Ch_4) were computed. For the voxel-wise FC differences between groups, a two-sample *t*-test was performed on the individual maps in a voxel-by-voxel manner. To examine linear relationships with sleep and mood measures, Pearson correlations were calculated between FC alterations and sleep and mood measures, respectively.

**Results:**

The ID group showed significantly decreased FC between the medial superior frontal gyrus and Ch_123 compared to HC. However, increased FC between the midbrain and Ch_4 was found in ID based on the voxel-wise analysis. The correlation analysis only revealed that the altered FC between the midbrain with Ch_4 was significantly negatively correlated with the self-rating anxiety scale.

**Conclusion:**

Our findings of decreased FC between Ch_123 and medial superior frontal gyrus and increased FC between midbrain and Ch4 suggest distinct roles of subregions of BF underlying the neurobiology of ID.

## Introduction

Insomnia Disorder (ID) is predominantly characterized by difficulties in initiating sleep, remaining sleep or early morning awakenings for at least three times per week over at least 3 months. Patients with ID are generally accompanied by impaired daytime function, mood disruption and cognitive impairments (1–3). Moreover, it increases the risk of accidents and impacts negatively the quality of life, and social productivity (4, 5). ID is a common sleep-wake disorder and is often considered to be related to central nervous system hyperarousal and increased cortical activation (6, 7). Previous animal studies have suggested that the cholinergic basal forebrain (BF) plays an important role in sleep-wake regulation and is implicated in cortical arousal and activation (8, 9). Moreover, the cholinergic BF is reported to be active during both wakefulness and rapid eye movement (REM) sleep but silent during non-REM (NREM) sleep (10), and the activation of cholinergic neurons enhances arousal, attention, and memory (11–16). Optogenetic activation of cholinergic BF causes animals to transition from slow wave sleep to wake (17). These previous animal findings suggest that BF might play a key role in understanding the underlying neuronal circuit for ID.

Most previous human studies that explored the role of BF in the sleep-wake cycle or ID are based on invasive positron emission tomography (PET) imaging techniques. Using H2(15)O-PET to evaluate the dynamic changes in cerebral blood flow (CBF) throughout the sleep-wake cycle in 37 normal male volunteers, one study found that BF showed profound deactivations during slow-wave sleep and reactivations during REM sleep (18). Nofzinger et al. reported insomnia patients showed a smaller decline in relative global cerebral glucose metabolism from waking to sleep states in BF compared to healthy controls (7). Moreover, the same research group further found that depressed patients with severe insomnia symptoms showed increased activation in the BF from waking to the REM sleep state (19). More importantly, Kajimura et al., found that CBF in the BF was lower during non-REM sleep when subjects were given triazolam (a short-acting benzodiazepine) than when they were given a placebo based on the H2(15)O-PET imaging (20). The finding suggests that the hypnotic effect of the benzodiazepines may be mediated mainly by the deactivation of the BF system. Therefore, these previous PET studies have shown that BF is involved in the sleep-wake cycle and is closely related to sleep disorders. However, it is not well established regarding the underlying neuronal mechanism of BF in ID as revealed by non-invasive fMRI techniques.

Resting-state fMRI (rs-fMRI) is a useful non-invasive imaging technique to measure spontaneous neural activity and is crucial for uncovering the intrinsic brain functional architecture. Functional connectivity (FC) analyses, using rs-fMRI to measure synchronous low-frequency brain activity fluctuations, have attracted increasing attention in the study of intrinsic neuronal properties of the brain in ID (21–24). However, non-invasive investigation of BF function has proven challenging due to difficulty in localizing with fMRI. A cytoarchitectonic atlas of the BF generated using post-mortem brains (25) has partially improved these issues. Currently, there is only premilitary evidence regarding the intrinsic FC alterations of BF in ID (26). The BF is important in the production of acetylcholine and is distributed widely to the cortical and limbic structures. Mesulam et al., identified four overlapping magnocellular groups within the basal forebrain BF and described four cholinergic cell groups Ch1–Ch_4 (27). More recently, Fritz et al., found that BF is functionally organized into two subregions that largely follow anatomically defined boundaries of the medial septum and diagonal band of Broca (MS/DB, Ch1-3) and nucleus basalis of Meynert (NBM, Ch_4) using the FC of BF based on rs-fMRI technique (28). Therefore, given that the BF is a heterogeneous structure with different subregions, we focused on exploring the distinct FC alterations of BF subregions in ID in this study.

To this end, we used voxel-based FC of BF subregions to investigate intrinsic spontaneous neuronal activity alterations in a large sample of ID patients (*n* = 102) and HCs (*n* = 96) who completed sleep and mood relate questionnaires. Additionally, we further explored the associations of sleep and mood measures with FC alterations. We hypothesized that Ch_123 and Ch_4 showed distinct patterns of FC alterations in patients with ID compared to HC. In addition, the altered FC of BF subregions correlated with sleep or mood-related symptoms in ID.

## Materials and methods

### Participants

The current study was approved by the ethics committee of Guangdong Second Provincial General Hospital. Written informed consent was obtained from each participant. The patients with ID were recruited in the Department of Neurology at Guangdong Second Provincial General Hospital. The diagnostic criteria for patients with ID were determined by an experienced neurologist, based on the criteria of the Diagnostic and Statistical Manual of Mental Disorders, version 5 (DSM-V). The specific criteria for each patient with ID are as follows: (i) self-complaint of difficulty in falling asleep, staying asleep, or waking up and being unable to fall back to sleep; (ii) symptoms occurring at least three times a week for at least 3 months; (iii) sleep problems occurred despite adequate opportunities to sleep; and (iv) no other sleep disorders (e.g., obstructive sleep apnea, sleep-related movement disorders), mental disorders, substance use, serious organ diseases. Moreover, the patients were free of any psychoactive medication for at least 2 weeks before and during the study.

All participants completed the Pittsburgh Sleep Quality Index (PSQI), the insomnia severity index (ISI), the self-rating depression scale (SDS), and the self-rating anxiety scale (SAS) to evaluate the sleep quality and mood status of ID patients. We excluded four subjects from further examination due to their depression scores (SDS > 73) or severe anxiety scores (SAS > 69). After these tests, each patient underwent rs-fMRI and structural MRI scanning. Three patients were excluded since T2-weighted fluid-attenuated inversion-recovery MR images for these patients showed abnormal hyperintense signals. Finally, 102 patients with ID were included in the study.

We also included 96 HC participants from the local community *via* advertisements. Sleep and mood questionnaires were also completed for all participants in the HC group. The inclusion criteria for participants in HC were as follows: (i) good sleep quality and an ISI score < 7; (ii) no psychiatric or neurologic diseases; (iii) no brain lesions or prior substantial head trauma, verified by conventional T1- or T2-weighted fluid-attenuated inversion-recovery MR imaging. None of the participants in the HC group was excluded from further analysis.

### MRI data acquisition

MRI data acquisition for each subject was performed on a 3.0-T MRI scanner (Ingenia; Philips, Best, Netherlands) at the Department of Medical Imaging at Guangdong Second Provincial General Hospital. Each subject underwent both the rs-fMRI and high-resolution anatomical data scanning. The rs-FMRI data were acquired using an echo-planar imaging (EPI) sequence with the following sequence parameters: TR/TE = 2000 ms/30 ms, flip angle: 90°, field of view = 224 × 224 mm^2^, resolution = 64 × 64 matrix, number of slices = 33, slice thickness = 3.5 mm with a 0.7 mm gap, a total of 240 volumes, and an acquisition time of approximately 8 min. To determine the cooperation of the participant during the MRI scanning, each subject was asked whether they fell asleep and opened their eyes during scanning after MRI scanning. We rescanned the subject to obtain new rs-MRI data if a participant could not follow the instruction.

The high-resolution anatomical images were acquired using the following parameters: TR/TE: 8 ms/4 ms; flip angle: 8°; 256 × 256 matrix, field of view: 256 × 256 mm^2^; voxel size: 1 × 1 × 1 mm^3^; and 185 sagittal sections without gaps, covering the whole brain.

### Resting-state-fMRI data preprocessing

The rs-fMRI datasets were pre-processed based on Data Processing and Analysis for Brain Imaging (DPARSF) toolbox, version 4.3.^1^ The first 10 EPI volumes for each participant were discarded for transient signal changes before magnetization reached a steady state. The head motion of all participants in the present study meets the criteria of translation < 1 mm or rotation < 1 degree in any direction as well as volume-to-volume mean frame-wise displacement (FD) < 2.0 m (29). There were no significant differences in the maximum (*p* > 0.05) or mean framewise displacement (*p* > 0.05) of head motion profiles between the two groups. Individual T1 structural images were segmented and the DARTEL toolbox was used to create a study-specific template for accurate normalization. The functional images were co-registered to the structural images and normalized into standard MNI space. To reduce the effects of confounding factors on the BOLD signal in gray matter, the signal of white matter, cerebrospinal fluid signals, as well as the Friston 24 parameters of head motion (30), were then regressed out from the time course of each voxel. Global signal regression was not regressed in the current study considering that it is a controversial preprocessing option in rs-fMRI analyses (31, 32) and BF is proven to regulate the global signal (33). Finally, temporal band-pass filtering (0.01–0.1 Hz) was adopted.

### Voxel-based functional connectivity analysis of basal forebrain subregions

**FIGURE 1:**
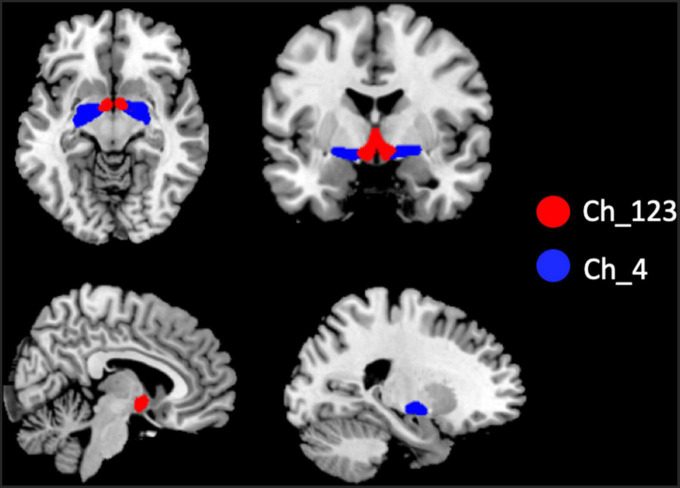
Illustration of the locations of basal forebrain (BF) subregions. The red color represents Ch_123; the blue color represents Ch_4.

**FIGURE 2:**
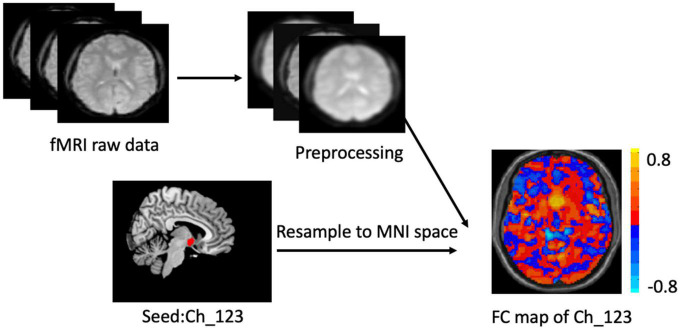
Flow chart of the seed_based functional analysis. Illustration of Ch-123 based functional connectivity (FC). Warms color in the FC map represents voxels with positive correlation with Ch_123, while cold colors represent voxels with negative correlation with Ch_123.

The two BF subregions, Ch_4 and Ch_123 covering both left and right hemispheres (Figure 1), were generated from the BF probabilistic maps in the SPM Anatomy toolbox (25, 34). The reasons we chose to use the BF probabilistic maps are as follows. First, the probabilistic cytoarchitectonic map of BF has been widely used in previous studies (35–38); Second, a recent study revealed that BF is functionally organized into two subdivisions that largely follow anatomically defined boundaries of Ch_123 and Ch_4 (28). The two seed regions were resampled to MNI space, in correspondence with the normalized rs-fMRI data. FC analysis of the two seed regions was conducted in a voxel-wise manner. For each seed region, voxel-wise FC maps were calculated by correlating voxel-wise the mean time series across a certain BF subnucleus with all time series across the brain. To assure the normality of FC correlation coefficient maps, the correlation coefficients were converted to *z*-values using Fisher’s transformation. Finally, the obtained z-maps were smoothed with a 6-mm full width at half maximum (FWHM) isotropic Gaussian kernel. The flow chart of the seed-based FC analysis was shown in Figure 2.

### Statistical analysis

A two-sample *t*-test was used to compare demographic data and neuropsychological tests between the HC and ID groups. The chi-squared test was used to examine sex distribution. For the voxel-wise FC differences between groups, a two-sample *t*-test was performed on the individual maps in a voxel-by-voxel manner. We used two cluster-forming thresholds to determine the significant results in this study. One is the stringent analysis using *p* < 0.05, Gaussian random field (GRF) corrected at the cluster level, based on a voxel-level threshold *p* < 0.001. The other one is a more liberal analysis using *p* < 0.05, GRF corrected at the cluster level, based on a voxel-level threshold *p* < 0.01. Pearson correlations were calculated between mean FC values in clusters showing significant group differences between ID and HC and sleep or mood-related measures, respectively.

## Results

### Demographic and neuropsychological characteristics

The demographic and neuropsychological characteristics of the HC and ID groups are shown in Table 1. There were no significant group differences in age, gender, education level, and BMI (all *p* > 0.05). As expected, the scores in all the sleep measures (PSQI, ISI), and mood measures (SAS, SDS) in the ID group are significantly higher than those in the HC group.

**TABLE 1:** Demographic and clinical characteristics between insomnia disorder (ID) and healthy controls groups.

Characteristic	ID (*n* = 102)	HC (*n* = 96)	Statistics	*P-*value
Age (years)	44.66 ± 15.42 (21–65 years)	44.26 ± 12.62 (24–65 years)	0.20	0.84
Gender (F/M)	76/26	64/32	1.47	0.23
Education (years)	13.32 ± 3.12	14.03 ± 3.85	–1.43	0.16
BMI (kg/m^2^)	21.69 ± 3.09	21.87 ± 2.51	–0.43	0.67
FD (mm)	0.10 ± 0.09	0.09 ± 0.04	0.830	0.13
PSQI	12.73 ± 3.57	2.67 ± 0.29	28.38	<0.001
ISI	15.62 ± 5.76	2.79 ± 1.00	21.52	<0.001
SAS	49.21 ± 11.28	33.46 ± 2.69	13.32	<0.001
SDS	52.91 ± 12.00	33.16 ± 1.52	16.00	<0.001

ID, insomnia disorder; HC, healthy control; PSQI, Pittsburgh Sleep Quality Index; ISI, insomnia severity index; SAS, self-rating anxiety scale; SDS, self-rating depression scale. Age range of the two groups is given in the brackets.

### Distinct functional connectivity alterations of basal forebrain subregions

**FIGURE 3:**
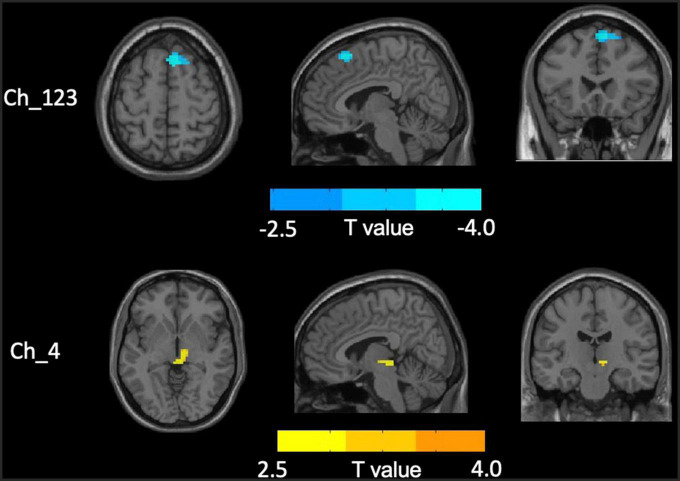
Distinct functional connectivity (FC) alterations of basal forebrain (BF) subregions in insomnia disorder (ID) compared to healthy control (HC). The figure represents decreased FC between medial superior frontal gyrus and Ch_123 as well as increased Ch4-midbrain FC in ID group compared to HC. The color bar represents the height of suprathreshold *t*-values.

No significant differences in any of the FC of subregions of BF were shown between ID and HC using the stringent statistical threshold. Under the liberal threshold, voxel-wise Ch_4-FC analysis revealed ID group showed significantly decreased FC between medial superior frontal gyrus and Ch_123 compared to HC (Table 2 and Figure 3). However, increased FC between midbrain and Ch4 was found in ID compared to HC based on the voxel-wise analysis (Table 3 and Figure 3).

**TABLE 2:** Brain region showing decreased functional connectivity with Ch_123 in insomnia disorder.

Brain region	Voxels	MNI			*T*-value
		x	y	z	
Frontal_Sup_Medial_R	60	6	24	60	4.340

R, right; MNI, Montreal Neurological Institute.

**TABLE 3:** Brain region showing increased functional connectivity with Ch_4 in insomnia disorder.

Brain region	Voxels	MNI			*T*-value
		x	y	z	
Midbrain_R	31	3	–27	–3	4.02

R, right; MNI, Montreal Neurological Institute.

### Relationships between alterations of basal forebrain subregions and clinical variables

**FIGURE 4:**
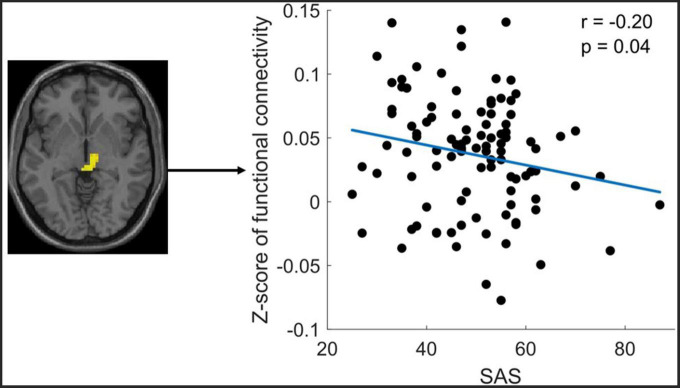
Significant correlation between functional connectivity (FC) of Ch4-midbrain and self-rating anxiety scale (SAS). The figure shows the significant negative correlation between the z-scores of the FC between Ch4-midbrain and anxiety score. The mean Z-Score of the FC was extracted from the significant cluster of the midbrain.

The correlation analysis only revealed that the altered FC between the midbrain with Ch_4 was significantly negatively correlated with the SAS (Figure 4).

## Discussion

In the current study, we used rs-fMRI datasets with voxel-based FC analysis to investigate FC alterations of BF subregions in ID. Our findings showed distinctly alterations of FC in the subregions of basal forebrain in ID, characterized by reduced FC between medial superior frontal gyrus and Ch_123 as well as increased FC between midbrain and Ch_4. In addition, the altered FC between the midbrain and Ch_4 correlated negatively with the SAS in ID. This study provided *in vivo* evidence for distinct alterations of FC of BF subregions in ID based on large sample size and may have important implications for understanding the neural mechanisms underlying symptoms and cognitive impairments in ID.

Our result showed reduced FC between medial superior frontal gyrus and Ch_123 in ID compared to HC. The medial superior frontal gyrus, especially the right side, has been considered to be a site of convergence of the dorsal and ventral attention networks (39). Moreover, it is also implicated in working memory, movement, and cognitive control (40, 41). Previous rs-fMRI studies have revealed the abnormal functional activity of the superior frontal gyrus from the regional level in ID, as measured by local connectivity metrics like Regional Homogeneity (ReHo) and Amplitude of Low-Frequency Fluctuations (ALFF) (42, 43). In addition, the global FC alterations of superior frontal gyrus were also reported by previous studies in ID, as measured by the seed-based FC and independent component analysis (ICA) (22, 23). Moreover, one structural neuroimaging study found a reduced gray matter volume of the parietal cortex in insomnia disorder compared to healthy controls (44). A decrease in cortical thickness in the bilateral parietal cortex has also been reported after 23 h of acute sleep deprivation (45). The BF has widespread cholinergic projections and is considered as a major neuromodulatory hub for brain regions supporting cognition, including attention, memory, and spatial navigation (6, 46–48). A recent human study using the rs-fMRI technique reported that there is specific connectivity between the Ch_123 and medial frontal gyrus in healthy young adults using the rs-fMRI technique (6). ID patients are normally characterized by deficits in working memory tasks (49) and several attentional processes (1). Therefore, the reduced FC between the Ch_123 and medial superior frontal gyrus may be the underlying neuronal circuit for cognitive impairments in ID.

Increased FC between midbrain and Ch_4 in ID compared to HC was found in this study. The enhanced FC between Ch_4 and midbrain suggests an elevated neuronal synchronization between the two regions. *Via* the monosynaptic anterograde tracing system, Zheng et al. found that cholinergic neurons in the NBM mainly projected to the midbrain and isocortex (50). The midbrain is a component of the ascending reticular activating system (ARAS), which plays a key role in arousal and sleep (51–53). Nofzinger et al. found that glucose metabolism in wake-promoting ARAS was increased in insomnia patients and ARAS hypermetabolism heightens emotional activities and produces an arousal state and insomnia (7). Furthermore, increased ALFF in the midbrain was reported in the insomnia patients compared to healthy controls (54). Therefore, the increased FC between the NBM-midbrain in our study may provide further evidence to support the theory of hyperarousal underlying the neurobiology of insomnia patients. Furthermore, the current study’s correlation analysis showed a significant negative correlation between the NBM-midbrain connectivity and the SAS score. This correlation result was supported by recent studies, which have reported that the midbrain has been critically implicated in anxiety-like behaviors (55, 56). The negative correlation in the current study may indicate that the increased NBM-midbrain connectivity reflects a compensatory mechanism to combat the anxiety problem in the ID patient.

There are several limitations in the current study. First, we only conducted the limited sleep-related variables using the sleep-related scales, the objective and comprehensive polysomnography was not employed. This could possibly explain the missing relationship between the altered FC of BF with the sleep-related variables. Further studies are needed to elucidate this research question. Second, cognitive tests were not included in this study. It’s better to use a battery of cognitive tests to comprehensively understand the specific correlation between the altered FC of BF and the cognitive functions of ID. Finally, the current study was cross-sectional, precluding inferences about the longitudinal alterations of the altered FC of BF over time.

In summary, our study based on a large sample size provides an important addition to previous literature, namely, by providing evidence for decreased FC between Ch_123 and medial superior frontal gyrus as well as increased Ch_4-midbrain FC in ID. The FC alterations of the BF subregions may reflect the abnormal and specific BF subregion neurotransmitter projections in ID. The distinct FC alterations of subregions of BF may indicate different roles of subregions of BF underlying the neurobiology of ID.

## Data availability statement

The raw data supporting the conclusions of this article will be made available by the authors, without undue reservation.

## Ethics statement

The studies involving human participants were reviewed and approved by Ethics Committee of Guangdong Second People’s Hospital. The patients/participants provided their written informed consent to participate in this study.

## Author contributions

GJ and SL contributed to the design of the study. YF, ML, and HW contributed to the data acquisitions. SL and YF contributed to the data analysis. GJ, SL, YF, ML, TW, and HW contributed to the interpretation of the results. All authors contributed to the manuscript preparation and revision and approved the final version of the manuscript.

## Footnotes

1
http://rfmri.org/DPARSF
